# Modeling Magnetostrictive Transducers for Structural Health Monitoring: Ultrasonic Guided Wave Generation and Reception

**DOI:** 10.3390/s21237971

**Published:** 2021-11-29

**Authors:** Gaofeng Sha, Cliff J. Lissenden

**Affiliations:** Department of Engineering Science and Mechanics, The Pennsylvania State University, University Park, PA 16802, USA; gps5348@psu.edu

**Keywords:** magnetostriction, guided waves, finite element modeling, dispersion curves, mode selection

## Abstract

Ultrasonic guided waves provide unique capabilities for the structural health monitoring of plate-like structures. They can detect and locate various types of material degradation through the interaction of shear-horizontal (SH) waves and Lamb waves with the material. Magnetostrictive transducers (MSTs) can be used to generate and receive both SH and Lamb waves and yet their characteristics have not been thoroughly studied, certainly not on par with piezoelectric transducers. A series of multiphysics simulations of the MST/plate system is conducted to investigate the characteristics of MSTs that affect guided wave generation and reception. The results are presented in the vein of showing the flexibility that MSTs provide for guided waves in a diverse range of applications. In addition to studying characteristics of the MST components (i.e., the magnetostrictive layer, meander electric coil, and biased magnetic field), single-sided and double-sided MSTs are compared for preferential wave mode generation. The wave mode control principle is based on the activation line for phase velocity dispersion curves, whose slope is the wavelength, which is dictated by the meander coil spacing. A double-sided MST with in-phase signals preferentially excites symmetric SH and Lamb modes, while a double-sided MST with out-of-phase signals preferentially excites antisymmetric SH and Lamb modes. All attempted single-mode actuations with double-sided MSTs were successful, with the SH3 mode actuated at 922 kHz in a 6-mm-thick plate being the highest frequency. Additionally, the results show that increasing the number of turns in the meander coil enhances the sensitivity of the MST as a receiver and substantially reduces the frequency bandwidth.

## 1. Introduction

Research and practice in using ultrasonic guided waves for the structural health monitoring (SHM) of plate-like structures has focused more on Lamb waves than shear-horizontal (SH) waves, perhaps because Lamb wave modes are easier to generate using an angle-beam transducer and gel couplant [1]. Moreover, both Lamb waves and SH waves have long-range nondestructive inspection capabilities. Although Lamb waves and SH waves are both dispersive and multimodal in general [2,3,4], the fundamental SH wave mode SH0 is nondispersive and propagates alone below the first cutoff frequency, which can lead to simpler signal processing for SHM. Generally, Lamb waves (having displacement profiles with two components) and their interaction with discontinuity (e.g., cracks and section loss) are more complicated [5] while SH waves (having displacement profiles of just one component) can exhibit simpler mode conversion behavior when interacting with a defect [6]. Another advantage of SH waves is that they do not leak into the fluid at the plate surface, while most Lamb wave modes are leaky, which causes significant attenuation when the plate is fluid loaded [7]. Magnetostrictive transducers (MSTs) generate SH waves well and can generate some of the Lamb wave modes that are more difficult to excite with angle-beam transducers; for example, the nonleaky S1 mode when the phase velocity is equal to the longitudinal wave speed [8].

Ultrasonic transducers for the generation and reception of guided waves in plates can be classified as either contact or noncontact. Contact transducers include piezoelectric angle beam transducers [1], piezoelectric wafer arrays [9], and piezoelectric fiber transducers [10,11]. For example, reference [11] developed a piezoelectric array that can simultaneously generate Lamb and SH waves using polyvinylidene difluoride (PVDF) film. Noncontact-type transducers include air-coupled transducers [12,13], electromagnetic acoustic transducers (EMATs) [14,15,16,17,18], and laser-ultrasound [19,20]. Since EMATs are cost-effective, versatile in design, and capable of high-temperature inspection [21,22], various applications have been reported in the literature. To name a couple, a single-sided EMAT was designed to generate the SH1 mode guided wave [14], and a double-sided EMAT was implemented to improve SH guided wave generation [15]. Furthermore, some studies chose a combination of different transducers for generation and reception [4,23].

Both Lorentz-force EMATs and magnetostrictive transducers (MSTs) belong in the EMAT category and can generate and receive guided waves. Normally, both types require a biased magnetic field and a conductive coil (e.g., spiral, meander and racetrack shapes) to impose a static magnetic field and a dynamic electromagnetic field [22]. MSTs need a magnetostrictive patch coupled to the waveguide, while Lorentz-force EMATs are applicable to electrically conductive waveguides. It is worth mentioning that Lorentz force EMATs and MSTs have also been used for nonlinear guided wave generation and reception to detect microstructure damage at an early stage [24,25,26]. The focus of this paper is on MSTs for SH waves and Lamb waves. Although a variety of guided wave MSTs have been developed for different applications [27,28,29,30,31], a fundamental understanding of MST performance is lacking. The underlying objective of this paper is to better understand and quantify the capabilities of magnetostrictive transducers for generating and receiving ultrasonic guided waves in plates through finite element simulations of the transducer/plate system.

This paper is structured as follows. First, the magnetostriction mechanism associated with MSTs is introduced. Then the configuration and design of a guided wave MST is described, after which the finite element model development for a guided wave MST is provided. It is helpful to define a coordinate system (please refer to the figures below) for the purpose of discussion. Thus, we consider waves propagating in a plate in the *x*-direction, where the plate thickness is in the *z*-direction. SH waves have a *u*_2_ (in-plane) displacement component and Lamb waves have *u*_1_ (in-plane) and *u*_3_ (out-of-plane) displacement components. Moreover, modeling results for various transducer characteristics are presented and discussed. Finally, conclusions are provided. 

## 2. Magnetostriction

Magnetostriction is a physical phenomenon describing the mechanical deformation of a material under an applied magnetic field. MSTs can convert magnetic energy into mechanical energy or vice versa. The material property quantifying the magnetostriction effect is called the magnetostriction coefficient. We use the sign convention where a positive sign means elongation, while a negative sign means compression. Ferromagnetic materials, including amorphous alloys [32] and elastomers [33], normally exhibit large magnetostriction coefficients and have practical applications as actuators and sensors. Engineering magnetostrictive materials with large magnetostriction coefficients are Terfenol, Galfenol, Remendur, and other FeCo alloys, and have been applied to ultrasound generators and receivers [27,28,34,35].

Like Lorentz-force EMATs [22], MSTs need a magnetic bias to impose a static magnetic field *H_s_* in the magnetostrictive patch and a coil with an excitation current to generate a dynamic magnetic field (perturbation) *H_d_* in the patch. The relative orientation of the static magnetic field and the magnetic perturbation determine the mode of the generated or received guided wave; specifically, *H_s_* and *H_d_* are parallel in Lamb wave MSTs, while they are perpendicular in SH wave MSTs. SH wave MSTs are based on the Wiedemann effect [36] for wave generation while Lamb wave MSTs rely on the Joule magnetostriction effect [37]. These MSTs can also receive guided waves due to the corresponding inverse effects.

**Figure 1:**
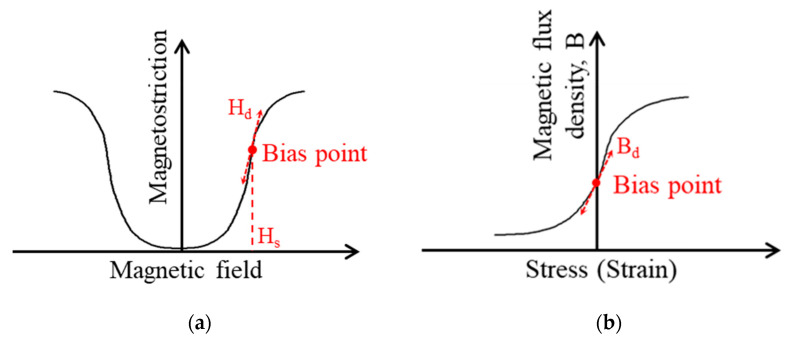
Linear model approximations for MST actuation and reception: (**a**) Linear approximation in actuation; (**b**) linear approximation in reception.

Generally, the magnetostriction behaves nonlinearly based on the magnetic field [37,38], but it has a nearly linear relationship with the dynamic magnetic field in MSTs because the dynamic perturbation is much smaller than the static magnetic bias (Figure 1a). Similarly, a linear approximation can be applied for reception by an MST (Figure 1b). Under the linear model assumption, constitutive equations for a magnetostrictive material are [39,40,41]. 

Stress–magnetization relationship:

(1) $$\begin{array}{c} \sigma_{ij}=c_{ijkl}^{H}\varepsilon_{kl}-e_{lij}^{\sigma }H_{l}\\ B_{i}=e_{ijk}\varepsilon_{jk}+\mu_{ij}^{\varepsilon }H_{j} \end{array}$$

Strain–magnetization relationship:

(2) $$\begin{array}{c} \varepsilon_{ij}=s_{ijkl}^{H}\sigma_{kl}+d_{kij}^{\sigma }H_{k}\\ B_{i}=d_{ijk}\sigma_{jk}+\mu_{ij}^{\varepsilon }H_{j} \end{array}$$

where $\sigma_{ij}$, $\varepsilon_{ij}$, $c_{ijkl}$, $s_{ijkl}$, and $\mu_{ij}$ are the tensors for stress, strain, stiffness, compliance, and magnetic permeability, $e_{ijk}$ and $d_{ijk}$ are the piezomagnetic coupling tensors, and $B_{i}$ and $H_{i}$ are the vectors for magnetic flux density and magnetic field. In COMSOL software, one can choose either the stress–magnetization relationship or the strain–magnetization relationship for a magnetostrictive material. Similar to the piezoelectricity tensor [42], the piezomagnetic tensor is a third-order tensor with the property $e_{ijk}=e_{ikj}$, $d_{ijk}=d_{ikj}$, and $e_{ijk}=d_{imn}{\left[s_{mnjk}^{H}\right]}^{-1}$.

As mentioned, the magnetostriction coefficient of a magnetostrictive patch depends on the static magnetic field intensity, but the explicit relationship is complicated [43]. Previous studies [44,45] strived to develop analytical models quantifying this relationship; however, these models, including the classical Yamamoto model [45], still suffer from limitations [46,47]. For simplicity, a piezomagnetic tensor with constant coefficients is adopted for this analysis. Moreover, a FeCo alloy patch with 0.1 mm thickness that has also been studied in References [24,48] is considered. The material properties of the FeCo alloy, such as the permeability and permittivity coefficients to be used in the COMSOL modeling, are taken from references [49,50].

## 3. Materials and Methods

The transducer and plate materials used in the finite element modeling as well as the methods used to prescribe the transducer geometry and loading are described in this section.

### 3.1. Magnetostrictive Transducers for Ultrasonic Guided Waves

**Figure 2:**
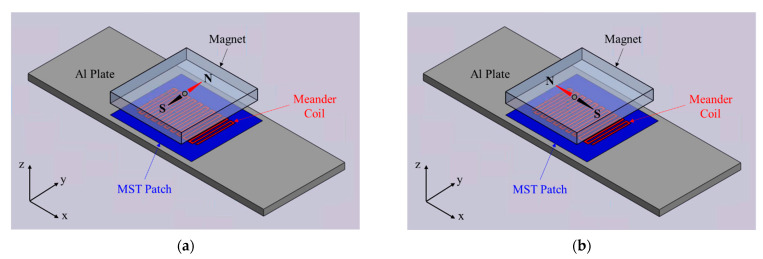
Magnetostrictive transducers for guided wave generation and reception in a plate: (**a**) SH wave MST; (**b**) Lamb wave MST.

Typically, an MST consists of a magnet, a meander coil, and a magnetostrictive patch. In practical applications, the magnetostrictive patches normally are pre-magnetized by swiping a permanent magnet or by applying a magnetic bias (Figure 2). The direction of the static magnetic bias *H_s_* must be perpendicular to the dynamic magnetic field *H_d_* in the magnetostrictive patch to excite SH waves (Figure 2a); Lamb waves will be generated when *H_s_* is parallel to *H_d_* (Figure 2b). The magnetostrictive patch is a native magnetostrictive plate or foil (e.g., remendur or galfenol), and it is coupled to the plate (comprised of any material) by an adhesive or other joining method. The magneostrictive patches could be cold-sprayed coatings [34]. The meander coil has a certain lift-off distance (which is beneficial for high-temperature inspection) and the turns in the meander should be located outside the footprint of the patch. Furthermore, a narrow bandwidth alternating current pulse (e.g., tone-burst) is applied to the meander coil to generate a guided wave in the magnetostrictive patch, which leaks into the plate and eventually forms SH waves or Lamb waves in the plate. In experiments, the reflections of guided waves from the ends of the patch need to be considered in the design; a tip to reduce the reflections from the ends of the patch is to trim the corners of the rectangle patch.

MSTs also can be used to generate and receive axisymmetric waves for pipe inspection, where the magnetostrictive patch, the meander coil, and the magnet have to be designed for the curvature of the pipe. Examples of MST designs for pipe inspection can be found in references [28,30].

**Figure 3:**
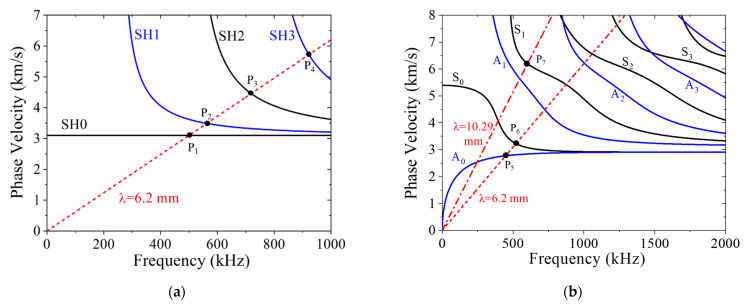
Phase velocity dispersion curves for guided waves in a 6-mm-thick aluminum plate with red activation line: (**a**) SH waves and (**b**) Lamb waves. The cross points are P1(500, 3.10), P2(563, 3.48), P3(713, 4.45), P4(922, 5.74), P5(447, 2.78), P6(520, 3.24), and P7(602, 6.19).

Unlike bulk waves, guided waves are multimodal and dispersive, which complicates the signal processing for SHM. Likewise, dispersion curve analyses and wave-structure computations are required for sensor selection to actuate and receive the desired guided wave mode. As indicated by previous studies [27,28,29], the possible SH-guided wave modes generated by an MST are determined by the coil geometry and the driving signal frequency. The SH wave and Lamb wave phase velocity dispersion curves are shown in Figure 3a,b, respectively. In this study, a fixed coil width and spacing giving a 6.2 mm wavelength will be considered. This is merely a convenience, as coils with different preferred wavelengths are easy to make. The actuation line (6.2 mm slope) is also shown in Figure 3a,b to illustrate which frequency is needed to generate each mode by such a coil. In Figure 3a, the crossing points corresponding to actuating the SH0, SH1, SH2, and SH3 modes are P1(500, 3.10), P2(563, 3.48), P3(713, 4.45), and P4(922, 5.74), where the frequency is in kHz (first number) and the phase velocity is in km/s (second number). In Figure 3b, the first two crossing points are P5(447, 2.78) and P6(520, 3.24) and they correspond to actuating the A0 and S0 Lamb modes. Exciting actuation points P1 to P6 will be simulated by COMSOL modeling. Another coil with a 10.29 mm wavelength will be used to actuate the nonleaky S1 mode where the phase velocity is equal to the longitudinal wave speed, whose actuation line is also shown in Figure 3b, with the actuation point P7(602, 6.19).

**Figure 4:**
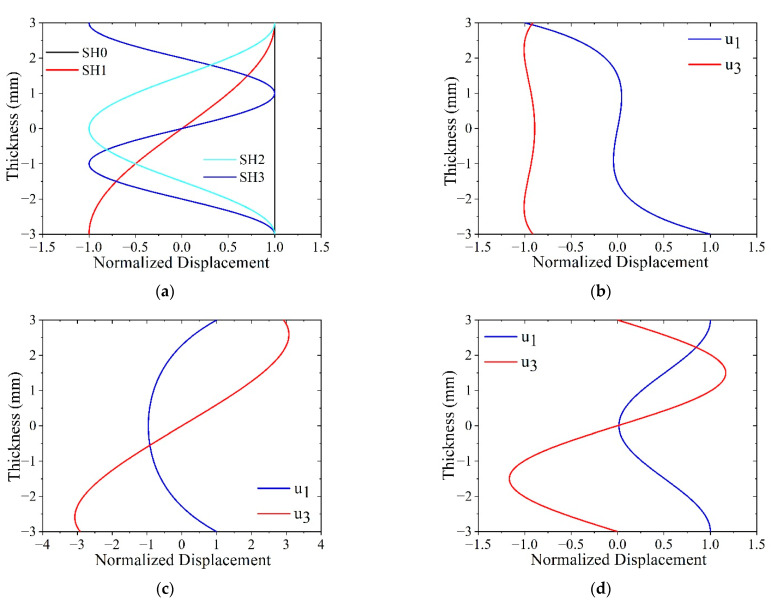
Wave structures, u_2_ for SH modes and u_1_ and u_3_ for Lamb modes: (**a**) u_2_ for SH0, SH1, SH2, and SH3; (**b**) A0 mode at P5(447, 2.78); (**c**) S0 mode at P6(520, 3.24); and (**d**) S1 mode at P7(602, 6.19).

To further demonstrate the difference between guided wave modes and facilitate later modeling results analysis, the wave structures of different modes are calculated and shown in Figure 4. The wave structures for wave modes SH0, SH1, SH2, and SH3 corresponding to P1, P2, P3, and P4 are plotted in Figure 4a. Wave structures of SH modes are sinusoidal functions for the *u*_2_ displacement component and are independent of frequency. Lamb wave structures are sinusoidal functions of frequency for the *u*_1_ and *u*_3_ displacement components [1]. The wave structure for the A0 mode at P5 is plotted in Figure 4b, and the wave structure for the S0 mode at P6 is plotted in Figure 4c, while the wave structure for the S1 mode at P7 is plotted in Figure 4d. The wave structures for Lamb waves were calculated using the semi-analytical finite element (SAFE) method [51].

### 3.2. Finite Element Model Development

**Figure 5:**
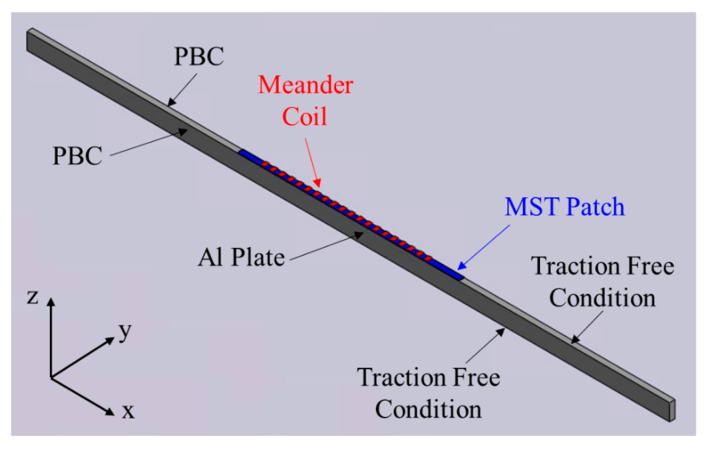
Transducer model schema for both SH waves and Lamb waves. The MST and the plate are surrounded by air. Periodic boundary conditions ensure planar wave propagation.

As discussed in Refs. [27,29], MSTs can be modeled using a linear model of the piezomagnetic tensor, and COMSOL Multiphysics software provides such a model for magnetostrictive materials [52]. In the finite element modeling using COMSOL only the meander coil, the magnetostrictive patch, and the waveguide (an aluminum plate in this study) need to be considered. Thus, the MST sketched in Figure 2 can be represented by the 3D COMSOL model shown in Figure 5, which is, in turn, discretized into quadratic tetrahedral elements. The schematic shows that only a thin strip of the MST/waveguide is modeled, with periodic boundary conditions (PBCs) applied to the lateral edges of the plate and the MST patch to ensure that the propagating waves are planar. The model contains only one element through the strip width. The PBCs include both the magnetic field and the displacement field. The top and bottom surfaces of the plate are traction free, and low-reflection boundaries are used at the ends of the model in the *x*-direction.

To model the MST and wave propagation in the plate in the time-domain, both Solid Mechanics and Magnetic Fields modules are used in the COMSOL Multiphysics software [52] and the physical fields of these two modules are coupled on the magnetostrictive patch. An alternating current excitation signal of 1 Ampere will be applied to the meander coil to generate a dynamic magnetic field in the FeCo patch, causing the transient displacement field. In turn, the vibrating FeCo patch excites guided waves in the Al plate. The center section of the meander coil having 10 turns (total length in the *x*-direction is ten wavelengths) is shown in Figure 5. Each adjacent coil has an opposite current direction (±*y*-directions). The specific excitation signal applied to the meander coil is a ten-cycle sinusoidal wave whose frequency is selected based on the actuation points P1–P7 and is given in Table 1. The turns in the meander coil are not explicitly modeled and the coil geometry remains the same for all cases analyzed. It would be interesting to determine in future modeling whether the coil turns generate nonuniformities in the wave field near the MST and whether such nonuniformities develop into waves. This future modeling effort will require the removal of the PBCs and a much larger modelled domain.

Since multiple modeling cases in the time domain will be performed in this study (actuation points P1–P7), the model input details for each case are given in Table 1. The same Al plate, FeCo magnetostrictive patch, and input current are used for all cases. The patch length is 74 mm for cases P1–P6 and it increases to 114.9 mm for case P7. The frequency of the ten-cycle driving signal for each case is also given in Table 1. The meander coil width and the spacing are both 1.55 mm for cases P1–P6 (corresponding to 6.2 mm wavelength) while they are about 2.57 mm for case P7. The lift-off of the meander coil is 0.1 mm for the whole study except where the impact of the lift-off distance is investigated. The material properties, such as density, permeability, permittivity, and electrical conductivity, used in COMSOL for the Al plate, FeCo patch, and meander coil are listed in Table 2. Since the MST and the plate are surrounded by air, its permeability, permittivity, and electrical conductivity are also used to model the electromagnetic field in the MST. It is worth mentioning that a tiny, but nonzero, electrical conductivity is necessary to form a closed current loop for the meander coil in this 3D model. In Section 4, different types of materials including titanium and stainless steel (SS) will be assigned to the plate to study the acoustic impedance effect, and the impact of an adhesive layer (having the same footprint as the MST patch) will also be investigated. Therefore, the material properties for Ti, SS, and adhesive are listed in Table 2 as well.

In time-domain modeling of the MSTs, the maximum mesh size for all the components, except air, is one-tenth of the guided wave wavelength (λ = *c_P_*/*f*) and the computation time step is one-hundredth of the wave period (1/*f*). The front surface is meshed first, and then the mesh is copied to the back surface, and finally all the domains are swept out.

The COMSOL model in Figure 5 is applicable to both SH waves and Lamb waves. To switch from SH waves to Lamb waves, we simply rotate the magnetization direction as shown in Figure 2. In the COMSOL model, the local coordinates of the piezomagnetic tensor need to be changed accordingly. Here the Bunge Euler angle rotation convention [54] was used in COMSOL to implement the piezomagnetic tensor rotation.

## 4. Results and Discussion

The results of the time-domain COMSOL simulations are presented here to demonstrate the influence of MST design variables and layout on guided wave generation and reception performance. In Section 4.1 the effect of individual design variables (e.g., magnetostrictive patch/plate acoustic impedance mismatch and coil lift-off) on SH0 mode generation will be discussed, then single-sided and double-sided MSTs are compared in Section 4.2. Furthermore, the reception bandwidth of an SH wave MST is analyzed in Section 4.3.

### 4.1. Effect of Individual MST Design Parameters on SH0 Mode Generation

In this section, a magnetostrictive patch bonded to a plate is actuated by a tone burst with a central frequency of 500 kHz to generate the SH0 wave propagating in the *x*-direction. The waveform is received at a point 167 mm from the MST. Specific model characteristics studied in this section are the FeCo patch thickness, FeCo patch/plate acoustic impedance mismatch, coil lift-off distance, adhesive layer thickness, and adhesive layer stiffness.

**Patch thickness effect.** Our modeling results indicate that the SH0 wave amplitude increases with FeCo patch thickness from 0.05–0.2 mm, remains constant at 0.3 mm, and then decreases thereafter. Glass et al. [34] report experimental results indicating that the SH0 wave amplitude is increased 6–10 dB by decreasing the patch thickness from 0.5 mm to 0.25 mm, when using air-sprayed commercially pure Ni patches. In both cases, the optimal thickness is nominally 0.25 mm.

**Figure 6:**
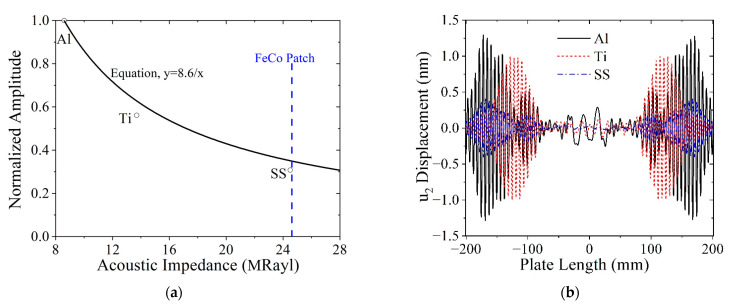
Acoustic impedance mismatch effect on MST performance: (**a**) SH0 wave amplitude decrease as a function of plate acoustic impedance; (**b**) u_2_ displacement field at 60 μs generated by the MST on different plates.

**Acoustic impedance mismatch effect.** For the normal incidence of waves on an interface, the acoustic impedance is used to assess transmission and reflection. Here we show that acoustic impedance mismatch is not the parameter to consider when assessing energy transfer from the FeCo patch to the plate. In the present case, the waves are actuated in the patch and propagate parallel to the interface. The continuity conditions at the interface require the wave energy to transfer to the plate. This is not a trivial analytical problem to solve and is beyond the scope of the current analysis, but as seen by the finite element analysis of various acoustic impedances, it is necessary to understand the energy transfer problem. We investigate how the acoustic impedance mismatch between the patch and the plate impacts the SH0 mode actuation by replacing the Al plate with Ti and SS. The patch and plate are assumed to be perfectly coupled. The acoustic impedance of the FeCo patch is 24.62 MRayl, while the acoustic impedance values of the Al, Ti, and SS are 8.6, 13.7, and 24.5 MRayl, respectively. The normalized SH0 wave amplitudes for Al, Ti, and SS plates are shown in Figure 6a, which indicates that the SH0 wave amplitude decreases as the acoustic impedance mismatch between the patch and plate decreases. In fact, the relationship between the SH0 wave amplitude and the acoustic impedance of the plate is inverse (y=α/x). The *u*_2_ displacement plotted along the length of the plate in Figure 6b shows the MST actuates waves that propagate in both directions and that there are multiple wave packets present, presumably the SH0 and SH1 modes (which will be analyzed subsequently).

**Figure 7:**
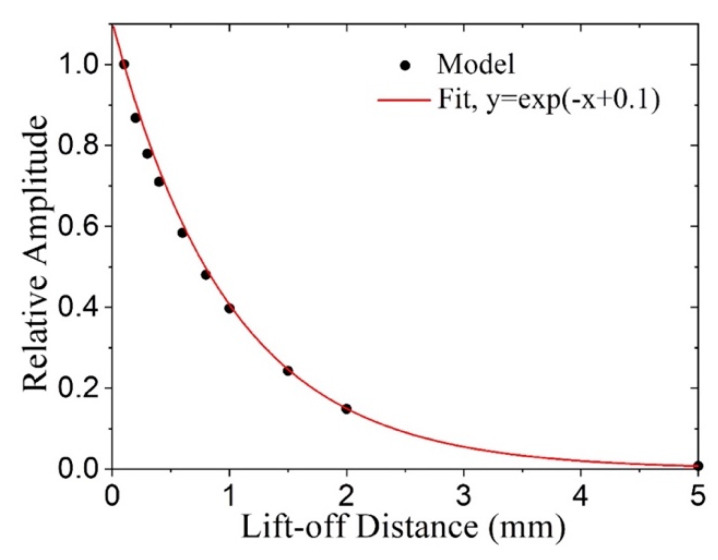
SH0 wave amplitude as a function of coil lift-off.

**Coil lift-off effect.** Lift-off, or an air gap between the coil and the magnetostrictive patch, is considered by varying the lift-off from its minimum value of 0.1 mm to almost 5 mm. Ideally, zero lift-off could be used as the reference, but it is unrealistic in experiments and would cause an issue in the model. Thus, the resulting SH0 amplitudes are normalized with respect to the lift-off of 0.1 mm. Figure 7 shows that the SH0 wave amplitude decays exponentially with lift-off distance, which is similar to Lorenz-force EMATs [22].

**Effect of adhesive stiffness.** Since the magnetostrictive patch is typically bonded to the plate with adhesive, it is instructive to study the effect of the adhesive stiffness on the MST performance. Results for 0.1-mm-thick adhesives with different Young’s moduli are compared. Each case is normalized with respect to the baseline case where the patch is ideally coupled to the plate without adhesive. The organic compound salol (phenyl salicylate) [53] is included in this analysis.

**Figure 8:**
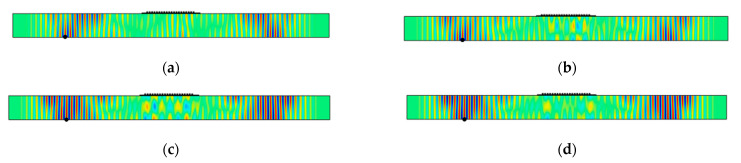

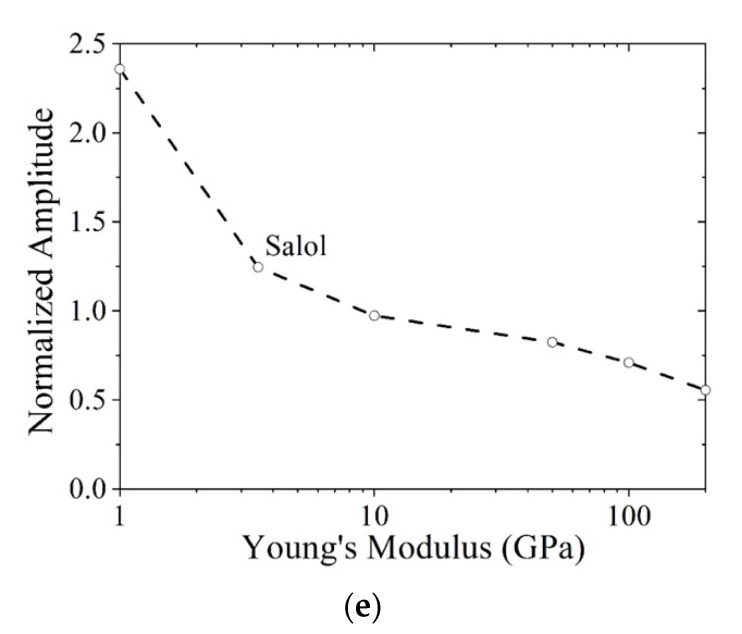
Normalized SH0 wave amplitude as a function of adhesive Young’s modulus. *u*_2_ distribution at 50 μs for: (**a**) *E* = 1 GPa; (**b**) *E* = 3.49 GPa; (**c**) *E* = 10 GPa; (**d**) *E* = 50 GPa; and (**e**) normalized SH0 wave amplitude as a function of adhesive Young’s modulus. Point marker in each plot (**a**–**d**) indicates the place where the maximum *u*_2_ displacement occurs.

The *u*_2_ displacement fields at 50 μs for different adhesive stiffnesses are shown in Figure 8a–d and the relationship between SH0 wave amplitude and Young’s modulus of the adhesive is shown in Figure 8e. In Figure 8e, the SH0 wave amplitudes are normalized by the baseline case and are observed to decrease with adhesive stiffness. In fact, a compliant adhesive increases the SH0 amplitude relative to ideal coupling. Observe from Figure 8a–d that the SH0 wavefield is not uniform through the plate thickness and that the spatial width of the wave packets increases from Figure 8a,d, even though the peak amplitude decreases. Finally, ringing underneath the MST patch takes place, and the most ringing occurs when Young’s modulus *E* = 10 GPa.

**Figure 9:**
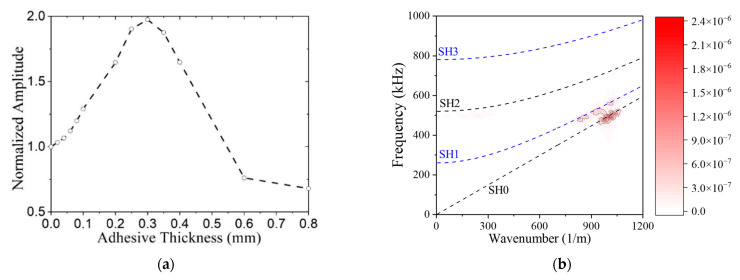

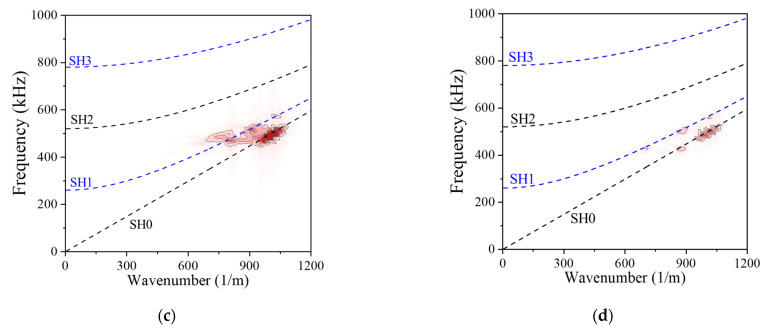
Effect of the adhesive thickness on SH0 wave amplitude and wave modes, (**a**) normalized wave amplitude as a function of adhesive thickness. Wavenumber spectra for MST with adhesive thickness of (**b**) 0.0 mm; (**c**) 0.3 mm; (**d**) 0.6 mm. (**b**–**d**) have the same color bar.

**Adhesive layer thickness effect.** In addition to the material properties of the adhesive affecting the MST response, its thickness will too. Adhesive thicknesses from 0 to 0.8 mm are considered, given the properties for salol. The influence of the adhesive layer thickness on the SH0 wave amplitude is shown in Figure 9a. As the adhesive thickness increases the amplitude first increases, reaches a peak, and then decreases. We found it surprising that the wave amplitude increased for adhesive thicknesses from 0–0.3 mm as traditional thinking holds that thin bond lines work best. Thus, we investigated the modal distribution by performing a 2D fast Fourier transform (FFT) and plotting the wavenumber spectrum on top of the dispersion curves for adhesive thicknesses of 0, 0.3, and 0.6 mm in Figure 9b–d. In each case, SH0 is the predominant mode, but the SH1 mode also propagates. Although there are higher intensities for the 0.3-mm-thick adhesive, the relative distribution between SH0 and SH1 appears to be roughly the same. Future work should investigate why the presence of a relatively compliant (*E* = 3.49 GPa) adhesive between the stiff plate (*E* = 70 GPa) and patch (*E* = 180 GPa) increases the amplitude of the actuated SH0 waves.

### 4.2. Comparison between Single-Sided and Double-Sided MSTs

Since double-sided Lorenz-force EMATs have shown superior capability in mode selection [15], this section considers both single-sided and double-sided MSTs to assess how effectively the MST preferentially actuates a prescribed mode at different frequencies. They are first compared for SH guided wave generation and then for Lamb wave generation.

#### 4.2.1. SH Wave MSTs

**Figure 10:**
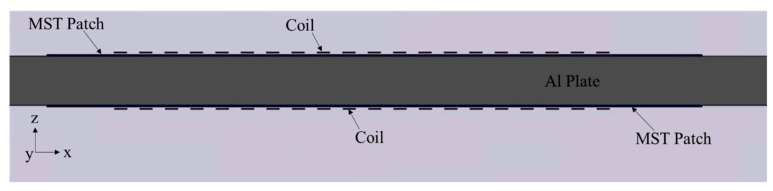
Double-sided MST/plate model includes the plate, two patches, and two 10-turn coils.

To enhance the SH waves in the plate, a double-sided MST, with the design shown in Figure 10, is investigated. Specifically, it is two separate MSTs perfectly bonded to an Al plate. The current directions in the upper coil and lower coil are properly selected to implement out-of-phase generation for antisymmetric modes and in-phase generation for symmetric modes. Such a design not only enhances the SH wave amplitude but also improves the wave mode control and could be especially powerful for generating higher-order SH guided waves.

**Figure 11:**
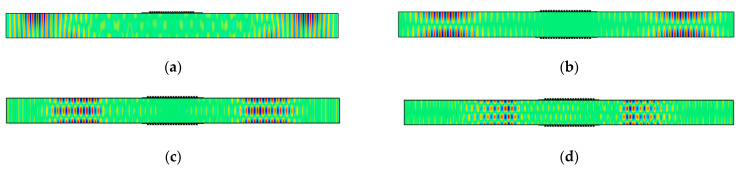
*u*_2_ displacement fields at 60 ms for (**a**) single-sided MST at 563 kHz, (**b**) double-sided MST at 563 kHz out-of-phase, (**c**) double-sided MST at 719 kHz in-phase, (**d**) double-sided MST at 922 kHz out-of-phase. The plots are stretched vertically for clarity.

**Figure 12:**
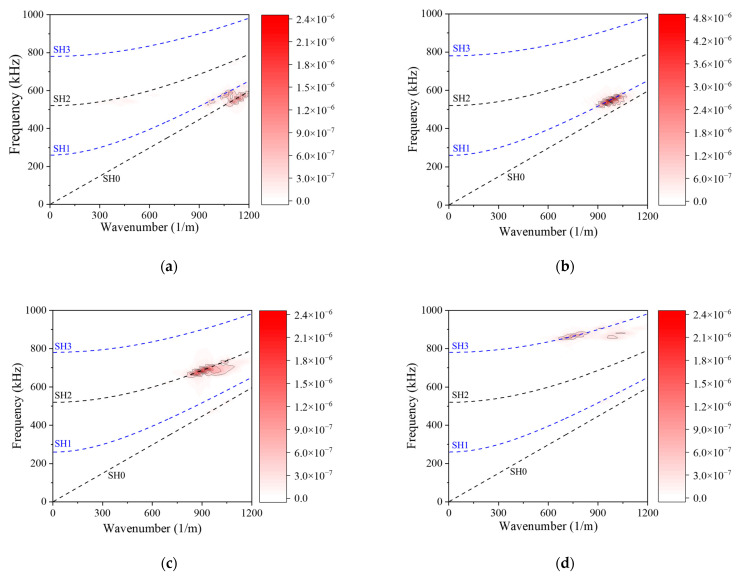
Wavenumber spectra acquired from wave fields in Figure 11: (**a**) Single-sided MST at 563 kHz, (**b**) double-sided MST at 563 kHz, (**c**) double-sided MST at 719 kHz, (**d**) double-sided MST at 922 kHz. The intensity scale in (**b**) is different from the others.

To demonstrate the capability of a double-sided MST for preferential mode actuation, model results for the antisymmetric SH1 mode (*u*_2_ displacement) using a double-sided MST with out-of-phase excitation at 563 kHz (actuation point P2) is shown in Figure 11b, while its counterpart for a single-sided MST with the same excitation signal is shown in Figure 11a. The SH1 wave structure (illustrated in Figure 4a) is antisymmetric for *u*_2_, meaning that *u*_2_ has equal magnitudes but opposite signs at equidistant points from the midplane. The double-sided MST provides an antisymmetric *u*_2_ displacement field, while the single-sided MST does not. The 2D FFT can be used to assess the modal content of the *u*_2_ displacement fields. 2D FFTs obtained from *u*_2_ data acquired along the bottom surface of the plate for single-sided (Figure 11a) and double-sided (Figure 11b) MST actuation are shown in Figure 12a,b, respectively. The dispersion curves for SH waves are superimposed on the wavenumber spectra to enable modal identification. The intensity range in Figure 11b is twice as large as the intensity range in Figure 11a and the *u*_2_ amplitude comparison for the SH1 mode is given in Table 3. More importantly, the double-sided MST predominantly excites the SH1 mode, while the single-sided MST appears to excite more of the SH0 mode than the SH1 mode. Thus, in addition to providing double the wave energy, the double-sided MST provides much improved control over which mode is generated.

The ability of double-sided MSTs to provide excellent mode control is further illustrated for the symmetric SH2 mode at 719 kHz (actuation point P3 with the wave structure shown in Figure 4a) in Figure 11c and Figure 12c. Likewise, double-sided excitation of the antisymmetric SH3 mode at 922 kHz (actuation point P4 with the wave structure shown in Figure 4a) is shown in Figure 11d and Figure 12d. In summary, the double-sided MST provided excellent mode control up to 922 kHz, which is an *fd* product of 5.5 MHz-mm. We did not investigate the upper limit of this capability. Giving the double-sided MST the same current at different frequencies led to the peak *u*_2_ displacement amplitudes of 2.0, 2.9, 1.4, and 0.8 nm for frequencies of 500 kHz (SH0), 563 kHz (SH1), 713 kHz (SH2), and 922 kHz (SH3), respectively. The amplitude increased from SH0 to SH1 and decreased thereafter for SH2 and SH3.

#### 4.2.2. Lamb Wave MSTs

The single-sided MST shown in Figure 5 and the double-sided MST shown in Figure 10 for SH wave actuation are compared in this section to evaluate their ability to actuate A0, S0, and S1 Lamb wave modes. In each case, the magnetostrictive patch is ideally coupled directly to the plate and the meander coil provides a 6.2 mm wavelength for A0 and S0 mode generation (actuation points P5 and P6) as it did for all the SH wave actuations, but a 10.29 mm wavelength is used for S1 mode generation (actuation point P7). As previously noted, the difference between the MST actuating SH waves and Lamb waves is the direction of the magnetic field bias as shown in Figure 2. In the COMSOL model, the magnetic field bias is determined by the magnetostriction tensor coefficients.

**Figure 13:**
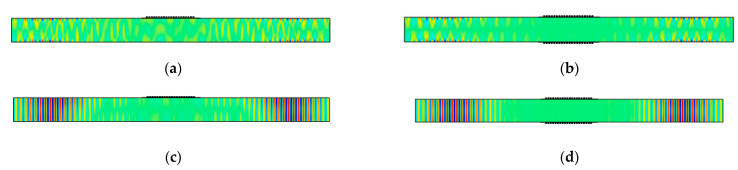
Snapshots at 60 μs of A0 Lamb wave mode actuation with 447 kHz signal: (**a**) *u*_1_ displacement from single-sided MST; (**b**) *u*_1_ displacement from double-sided MST out-of-phase; (**c**) *u*_3_ displacement from single-sided MST; (**d**) *u*_3_ displacement from double-sided MST out-of-phase. The plots are stretched vertically for clarity.

Model results for the single-sided MST and the double-sided MST with a 447 kHz excitation signal are shown in Figure 13. Since two displacement components, *u*_1_ and *u*_3_, exist in Lamb waves, the *u*_1_ and *u*_3_ displacement fields at 60 μs from the single-sided MST are shown in Figure 13a,c, respectively, while those from the double-sided MST are shown in Figure 13b,d. The *u*_1_ and *u*_3_ displacement fields for both MSTs have similar distribution patterns, namely *u*_3_ displacement has a uniform distribution across the plate thickness while the *u*_1_ displacement exhibits extrema on the top and bottom surfaces of the plate. These features agree with the wave structure of the A0 mode. However, the amplitudes of the *u*_1_ and *u*_3_ displacements from these types of MSTs are different and these values are listed in Table 3. The *u*_1_ and *u*_3_ amplitudes for the double-sided MST are roughly twice that of their counterparts for the single-sided MST for the A0 and S0 modes, which is also true for the *u*_2_ amplitudes for the SH0 mode. Note that the *u*_2_ amplitude for the SH0 mode is larger than the bigger of the *u*_1_ and *u*_3_ amplitudes for the A0 and S0 modes, but they are comparable. Thus, it appears that MSTs are only slightly more efficient generators of SH waves than Lamb waves.

**Figure 14:**
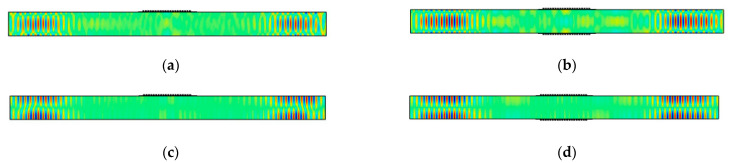
Snapshots at 80 μs of S0 Lamb wave mode actuation with 520 kHz signal: (**a**) *u*_1_ displacement from single-sided MST; (**b**) *u*_1_ displacement from double-sided MST in-phase; (**c**) *u*_3_ displacement from single-sided MST; (**d**) *u*_3_ displacement from double-sided MST in-phase. The plots are stretched vertically for clarity.

Furthermore, the single-sided MST and the double-sided MST in the models of Figure 13 are loaded with a 520 kHz excitation signal (actuation point P6) and the model results are shown in Figure 14. Particularly, the *u*_1_ and *u*_3_ displacement fields at 80 μs for the single-sided MST are shown in Figure 14a,c while displacement distributions for the double-sided MST are depicted in Figure 14b,d. According to the wave structure of the S0 mode in Figure 4c, the *u*_1_ and *u*_3_ displacement distributions in Figure 14 from either MST agree with the S0 mode very well, which indicates that a single-sided MST also excites the S0 mode well. The amplitudes of *u*_1_ and *u*_3_ displacements from both MSTs are listed in Table 3, and the *u*_1_ and *u*_3_ displacements from the double-sided MST are about twice those of the single-sided MST.

**Figure 15:**
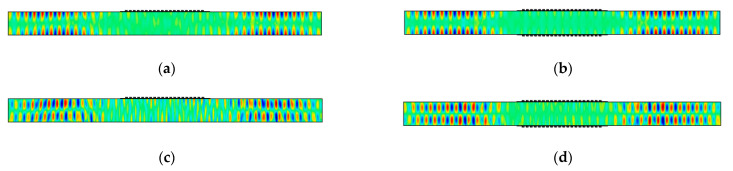
Snapshots at 40 μs of S1 Lamb wave mode actuation with 602 kHz signal: (**a**) *u*_1_ displacement from single-sided MST; (**b**) *u*_1_ displacement from double-sided MST in-phase; (**c**) *u*_3_ displacement from single-sided MST; (**d**) *u*_3_ displacement from double-sided MST in-phase. The plots are stretched vertically for clarity.

Finally, models for the single-sided MST and the double-sided MST with a 10.29 mm wavelength meander coil and a 602 kHz excitation signal (actuation point P7) are solved to demonstrate the MST excitability of S1 Lamb waves. The *u*_1_ and *u*_3_ displacement fields at 40 μs for the single-sided MST and the double-sided MST are given in Figure 15. The wave structures for both MSTs in Figure 15 are consistent with the S1 wave structure in Figure 4d, which indicates that MSTs are capable of generating S1 mode although the *u*_3_ displacements on the top and bottom surfaces of the plate are zero. The displacement distribution patterns of *u*_1_ and *u*_3_ for the single-sided MST are similar to these on the double-sided MST except that the antisymmetric *u*_3_ pattern in Figure 15c is not as obvious as its counterpart in Figure 15d. The amplitudes of the displacements for both cases are listed in Table 3. The amplitude of the *u*_1_ displacement from the double-sided MST is roughly 1.5 times that from the single-sided MST. Although the amplitudes of the *u*_3_ displacement for both cases are not zero on the surface (as in the wave structure), they are comparable to the noise level in the waveguide, e.g., the ringing underneath the MST patch.

### 4.3. Reception Bandwidth of SH0 Wave MSTs

The previous sections addressed guided wave actuation with an MST. In this section, we focus on using the MST as a receiver, and specifically on the reception bandwidth for an SH0 wave. As in Section 4.1, a single-sided MST ideally coupled to the plate is considered, and the meander coil is set to receive a 6.2 mm wavelength. The MST is used only to receive and the SH0 waves are generated through the application of ten sinusoidal cycles of the *u*_2_ displacement at the left end of the plate (the left side of the coil is approximately 33 mm from the displacement loading plane). The amplitude is 1 nm and its frequency varies from 250 to 1000 kHz to assess the bandwidth of the MST as a receiver. In all previous simulations, the meander coil had 10 turns, but in these simulations, it has 1–6 turns. The meander coil only receives voltage signals that are generated by the wave motion experienced by the magnetostrictive patch due to the inverse Wiedemann effect.

**Figure 16:**
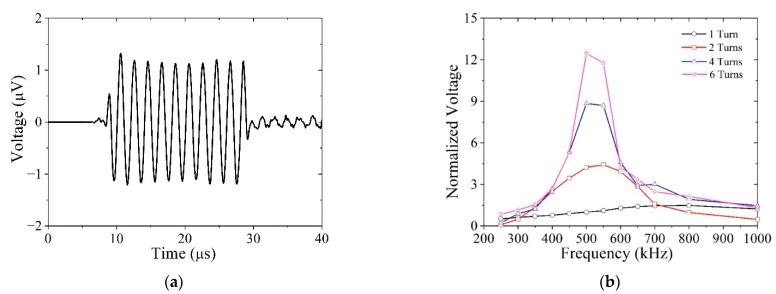
Reception bandwidth of SH wave MSTs with different numbers of coils: (**a**) A-scan signal showing the received signal for 500 kHz and a 1-turn coil (reference case); (**b**) normalized amplitudes of SH0 waves at different frequencies received by different coils.

The MST reception bandwidth is shown in Figure 16 by plotting the A-scan (amplitude-time) for the reference case and the received voltage as a function of the wave’s excitation frequency. We normalize the received voltage with respect to the reference case of an MST with a single turn in the coil and the SH0 waves propagating at 500 kHz. The one-turn coil provides a broadband response between 250 and 1000 kHz, but by using multiple turns the sensitivity of the MST is greatly enhanced at the intended operating frequency (500 kHz here). Six turns increased the received voltage by at least a factor of 12. The clipped peaks for four-turn and six-turn coils indicate that smaller increments in frequency are needed to fully characterize the peak response. Clearly, MSTs with six or more turns have a relatively narrow reception bandwidth at their design frequency. In explanation, we note that the functioning of an MST with a meandering coil is similar to that of a comb transducer, which acts like a filter in the wavenumber domain [1]. The more elements in the comb, or turns in the coil, the more narrowband the filter is. Likewise, the narrowband filter is more sensitive than the broadband filter. An example where the MST reception bandwidth is important is for nonlinear guided waves, which are sensitive to early stages of material degradation, where the amplitude of the second or third harmonic needs to be measured. In [24], the third harmonic of the SH0 waves is received with a coil with turns spaced at one-third the spacing used to generate the primary waves.

## 5. Conclusions

A multiphysics finite element model of a magnetostrictive transducer (MST) on a plate was created to study the effects of transducer characteristics on the actuation and reception of ultrasonic guided waves. MST design variables and architecture were investigated with respect to actuating shear-horizontal (SH) waves and Lamb waves for structural health monitoring. The sole difference between SH wave generation and Lamb wave generation is the magnetization direction in the magnetostrictive patch, which dictates the wave polarization. The results indicate that the same transducer given the same input parameters will generate SH waves with significantly different amplitudes in different materials, e.g., the amplitude in stainless steel is 30% that in aluminum for a FeCo patch. The thickness and stiffness of an adhesive layer between the patch and the waveguide also affect the wave amplitude. For the parameters we studied, a compliant adhesive is best, and the optimal thickness is around 0.3 mm.

A double-sided MST with in-phase excitation for symmetric wave modes and out-of-phase excitation for antisymmetric modes is much better at preferentially actuating a single mode at the design frequency, which is dictated by the turn spacing in the meander coil, which in turn dictates the wavelength. Each attempted preferential single mode actuation was successful up to 922 kHz (for the antisymmetric SH3 mode) in a 6-mm-thick plate. The A0 and S0 modes were well actuated, as was the nonleaky S1 mode (with zero out-of-plane displacement at the surface). Moreover, the MST generates more SH waves than Lamb waves with comparable amplitudes, although the SH wave amplitudes are a little higher.

Finally, the reception capability of an SH wave MST was investigated. The results showed that as the number of coil turns increases, the sensitivity increases, while the reception bandwidth decreases. The MST receiver acts essentially as a filter, and as the number of coil turns increases, the pass bandwidth decreases.

## Figures and Tables

**Table 1:** Component geometry and excitation signal frequency for different modeling cases.

Case	Plate Length × Thick	FeCo Patch Length × Thick	Meander Coil Length × Thick	Excitation Signal Frequency, *f* (kHz)
P1	402 × 6 mm	74 × 0.1 mm	62 × 0.1 mm	500
P2				563
P3				719
P4				922
P5				447
P6				520
P7	402 × 6 mm	114.9 × 0.1 mm	102.9 × 0.1 mm	602

**Table 2:** Material properties for each component in the MST/plate model.

Component	Density (kg/m^3^)	Relative Permeability	Relative Permittivity	Electrical Conductivity (S/m)	Elastic Constants: *E* = Young’s Modulus ν = Poisson’s Ratio
Al plate	2700	1	1	6 × 10^7^	*E* = 70 GPa ν = 0.33
FeCo patch *	8000	38	1	1 × 10^6^	Ref. [49]
Meander Coil	2700	1	1	6 × 10^7^	*E* = 70 GPa ν = 0.33
Ti plate	4420	1	1	7.4 × 10^5^	*E* = 113 GPa ν = 0.33
SS plate	8027	1	1	1.35 × 10^6^	*E* = 195 GPa ν = 0.3
Salol [53]	1230	1	1	100	*E* = 3.49 GPa ν = 0.42
Air	-	1	1	10	-

* Piezomagnetic constants: d_31_ = −60.3 pm/A, d_33_ = 125 pm/A, d_15_ = 318 pm/A.

**Table 3:** Comparison of displacements on the bottom surface of the plate for single-sided and double-sided MSTs.

Preferential Mode	Single-Sided MST			Double-Sided MST		
	*u*_1_ (nm)	*u*_2_ (nm)	*u*_3_ (nm)	*u*_1_ (nm)	*u*_2_ (nm)	*u*_3_ (nm)
SH1	0	1.23	0	0	2.88	0
A0	0.47	0	0.71	1	0	1.36
S0	0.34	0	0.65	0.58	0	1.09
S1	1.16	0	0.29	1.78	0	0.32

## Data Availability

The data in this study are available in the article.
